# Opera 2015 Project: Accurate Measurement Equipment for Earthquake Electromagnetic Emissions and Radio Seismic Indicator

**DOI:** 10.3390/s23052379

**Published:** 2023-02-21

**Authors:** Renato Romero, Luca Feletti, Claudio Re, Andrea Mariscotti

**Affiliations:** 1 Independent Researcher, Str. Luisetti 12, 10040 Cumiana, Italy; 2 Independent Researcher, 10053 Bussoleno, Italy; 3 Broadcasting Network Supervision, World Family of Radio Maria, 10100 Turin, Italy; 4 Department of Electrical, Electronic and Telecommunications Engineering, and Naval Architecture, University of Genova, 16145 Genova, Italy

**Keywords:** amplifier, antenna, earthquake, earthquake sensing, ELF band, electric field, magnetic field, radio seismic indicator, seismic activity, seismic precursor

## Abstract

Electromagnetic emissions from earthquakes are known as precursors and are of considerable importance for the purpose of early alarms. The propagation of low-frequency waves is favored, and the range between tens of mHz to tens of Hz has been heavily investigated in the last thirty years. This work describes the self-financed Opera 2015 project that initially consisted of six monitoring stations over Italy, equipped with electric and magnetic field sensors, among others. Insight of the designed antennas and low-noise electronic amplifiers provides both characterization of performance (similar to the best commercial products) and the elements to replicate the design for our own independent studies. Measured signals through data acquisition systems were then processed for spectral analysis and are available on the Opera 2015 website. Data provided by other world-known research institutes have also been considered for comparison. The work provides examples of processing methods and results representation, identifying many exogenous noise contributions of natural or human-made origin. The study of the results occurred for some years and led us to think that reliable precursors are confined to a short area around the earthquake due to the significant attenuation and the effect of overlapping noise sources. To this aim, a magnitude-distance indicator was developed to classify the detectability of the EQ events observed during 2015 and compared this with some other known earthquake events documented in the scientific literature.

## 1. Introduction

An earthquake (EQ) can be a source of electromagnetic waves, in particular at low frequency, with a favorable propagation through the crust to the surface, where ground-based stations are located. Attenuation, in fact, becomes significant as soon as the frequency reaches values of 100 Hz. Emissions are associated directly with the EQ or may precede it, sometimes by several weeks. These electromagnetic field emissions may be exploited as precursors (often named “radio precursors” or “radio seismic precursors”), able to anticipate seismic events. There are different opinions on the detectability of such precursors, with some authors asserting a worldwide detectability, at least for the most intense EQs, such as those with a Richter magnitude above 6 or so [1,2,3].

Trivially, if radio seismic precursors exist, they will be easily received near the EQ and if the EQ has high intensity. Since the possibility of propagation of a signal in the ground increases with the wavelength, it is most likely that if radio waves are generated at depth, then only those waves with the lowest frequencies will be able to emerge at the surface. Low frequencies imply long wavelengths and long Fresnel distances [4] so in most cases, the monitoring station will be in the reactive region.

A range of precursors have been reported and discussed as early as in the 1990s [5], in particular after the significant EQ event of Loma Prieta [6]: both electric and magnetic field changes in the lithosphere were identified, although the quality and quantity of measurements were limited until approximately the turn of the millennium; Uyeda et al. [7], for example, reported evidence of seismo-electromagnetic phenomena below the extremely low-frequency (ELF) range (3–30 Hz), corroborated by many other studies in the following years, as listed in [5]. The discussion on the reproducibility and effectiveness of such findings and on the identification of a set of reliable indicators of the electromagnetic type is still ongoing, possibly extending the range of electromagnetic phenomena to the total electron content (TEC) [8,9,10] or plasma anomalies [5], although there is the concomitant influence of radon ionization [11], as well pointed out in [8], and some previous studies concluded on the lack of statistically significant traces with the state-of-the-art measurements at that time [12,13,14].

The Opera 2015 project [15], a self-financed project, carried out systematic ELF monitoring during 2015 from six ground stations in Italy with the objective of collecting a wide range of electromagnetic (e.m.) signals and to post-process them by looking for precursors. Although the collective effort of data gathering was concentrated in 2015, the activity has continued in successive years. The collected data turned out to be useful for some recent scientific work thanks to their completeness [16].

Reported e.m. emissions are concentrated mainly at frequencies bracketing the Schumann resonance, SR (nominally at 7.83 Hz), located within the ELF interval, and then going down to some tens of mHz (as analyzed in [7]). Due to significant e.m. pollution caused by natural and human activity, sensitive instruments and sophisticated processing techniques are used: directional and sensitive magnetic solenoids, large electric field wire antennas, low-noise stable electronic circuits for buffering and amplification and a wide range of signal processing methods (e.g., spectral and fractal analysis, principal component analysis and direction finding).

In particular, magnetic field emissions can be measured with a significant directivity by means of induction coils (solenoids) [17,18,19]: a two-axes arrangement with orthogonal coils is able to detect the direction of arrival; the larger the solenoid (in terms of area and number of turns) and the larger the captured signal, although winding resistance and inductance increase (and thus the internal impedance). A length/diameter ratio larger than 10 favors directivity and is commonly used for search coils.

E.m. pollution can both affect the quality of recorded signals and provide false positive e.m. anomalies associated with precursors. The ELF band is characterized by a myriad of natural signals, such as geomagnetic pulsations, the mentioned Schumann resonances, solar storms and electrostatic discharges (in particular, around thunderstorms). We can add to these signals other disturbances of human origin, such as industrial processes employing large current intensity and following work cycles of seconds and fractions of a second, as well as electrified railways. We obtain thus a picture of a band populated by a large number of signals, differing from each other not only in terms of frequency and amplitude but also duration, intermittency and general, statistical behavior. A great effort is thus necessary to first remove all e.m. disturbance, often making signal acquisition at particular parts of the day impossible.

This paper describes, in Section 2, the Opera 2015 project and the instrumentation used at the various stations, going into some relevant details of the designed sensors and electronics; one of the main purposes of this paper is, in fact, enabling the reader to replicate (and improve) the instrumentation, increasing the number of existing stations and the chance of capturing significant e.m. phenomena to enlarge the dataset of available signals. Section 3 lists the most relevant EQ events that were recorded and have been used since then for analysis and comparison, exemplified in Section 4, which provides details of the spectral components and combined behavior of sensors’ signals. Section 5 then discusses the experience regarding the detectability of precursors for EQ events and presents the expression of an indicator that takes into account magnitude and distance to decide on the detectability of e.m. precursors.

## 2. Opera Project Description and Instrumentation

**Figure 1:**
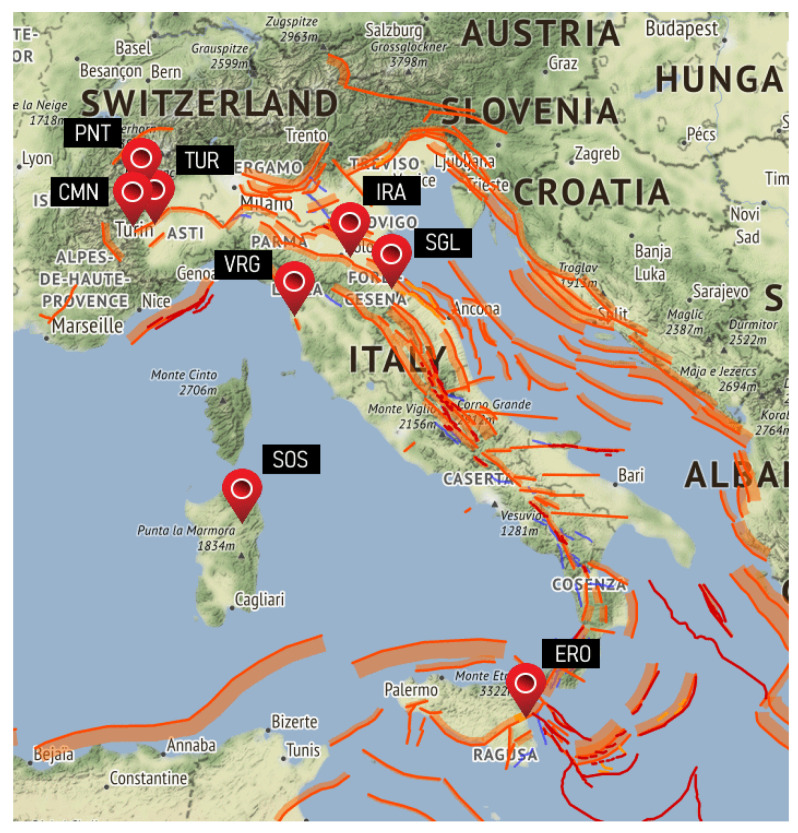
Location of monitoring stations over Italy: the six Opera 2015 stations are identified as CMN, PNT, TUR, IRA, SGL and ERO; the other two stations were added later, and they are SOS (for Sos Enattos in Sardinia) and VRG (for the EGO installation at Cascina near Pisa, in cooperation with the Virgo project).

The measurement campaign collected data from 14 sensors positioned in 6 different locations of high seismic activity. The monitoring stations were continuously active for a total of more than 110,000 recording hours during the project. A total of 15,532 EQs detected during 2015 by the Italian Institute of Geophysics and Volcanology were evaluated, so an average of 40 earthquakes per day. Six monitoring stations were active with various combinations of measurement instruments, as reported in Table 1 and graphically described in Figure 1.

Earth’s magnetic field parameters at the stations are then reported in Table 2: the dipole and quasi-dipole information [20] has been calculated using the model proposed by Emmert et al. [21] and available in [22]; the seven magnetic field parameters (declination, D, inclination, I, North oriented intensity, X, Eastern oriented intensity, Y, horizontal intensity, H, vertical intensity, Z, and total intensity, F, as main field components and secular variation, expressed as variation per year) have been calculated using the IGRF model [23,24]. For these calculations, the reference date was taken as 30 June 2015, so intermediate over the time span of project data.

Two of the former monitoring stations have recently ceased their activity (Rifugio Pontese, PNT, and Torino VLF, TUR), leaving Cumiana station in the Piemonte region, but two new stations have been added in two otherwise uncovered parts of Italy: Sos Enattos (SOS) in Sardinia, to host the Einstein Telescope for the study of gravitational waves, and Cascina, Pisa (VRG), at the Virgo center, the European Gravitational Observatory.

The instrumentation, succinctly listed in Table 1, is better described and characterized in the following.

### 2.1. Induction Coil

**Figure 2:**
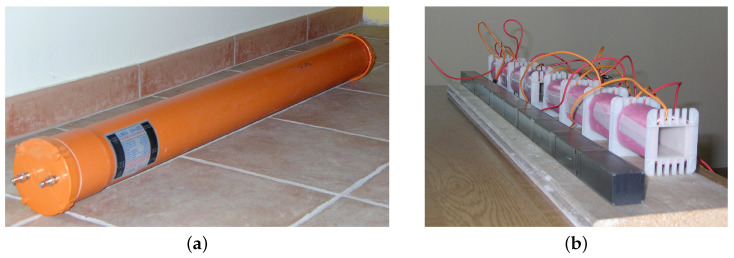
H-field solenoid with ferrite core: (**a**) final assembly; (**b**) construction detail with coil sections and ferrite rod.

The magnetic field sensor is an induction coil (solenoid), identified as ICS101 and developed as part of the OpenLab project (documented in the Opera 2015 website [15]). Contrary to other air coil arrangements [25] for the purpose of environmental measurements with a large angle and low directivity, the coil is not only significantly long but uses a high-permeability core rather than air. The coil is built around a composite core made of aligned high-permeability ferrite cores, commercially available for power converter applications. The material is Ferroxcube 3C90, and 10 ferrite bars (30 × 28 × 93 mm) were mounted next to each other for a total length of 93 cm. The ferrite core is longer than the overall winding, and two end sections are made of ferrite elements only, with the central eight providing the solenoid sections; the reason is to limit the end effects and better shape the magnetic field lines. The winding was divided into eight sections to lower the self-resonance: it consists of 96,000 turns of enameled copper wire for a total of 2.5 kg of copper and a length of more than 13 km of wire. The ferrite core and coils and the final assembly are shown in Figure 2.

Like all H-field antennas, this induction coil was externally shielded to attenuate E-field pick-up, using a tiny metallic grid arranged so as to break electrical continuity, avoiding a short-circuited turn impairing the solenoid operation. In [25], where the coil has a much smaller size, this was instead achieved by applying an insulated gapped copper strip on top of the circular winding, providing a dielectric layer to reduce stray capacitance terms toward the added shield.

**Figure 3:**
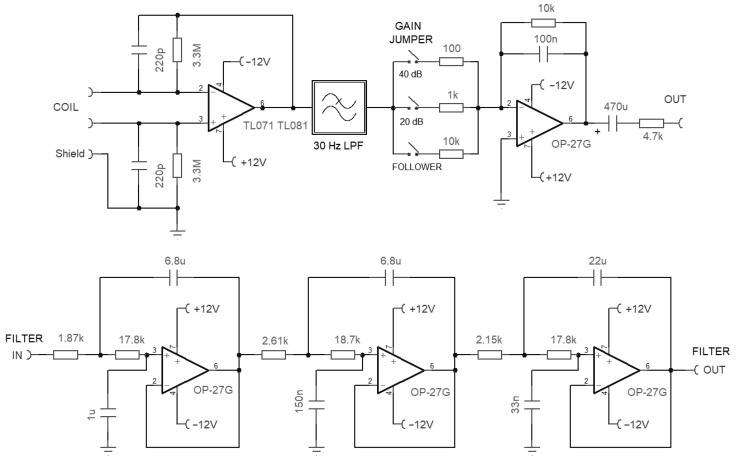
Schematic of the ICS101 large-gain amplifier for the induction coil, including the Chebychev low-pass filter (LPF).

The signal from the induction coil is conditioned by a custom-made large-gain amplifier, whose schematic is visible in Figure 3.

The coil amplifier is floating and has a symmetric structure, with the coil shield terminated on its signal ground. The coil current, rather than the coil voltage, is read on a virtual ground at the OA terminals by means of a transconductance amplifier circuit; this linearizes the frequency response avoiding the derivative effect. The derivative was instead exploited in [25] to increase the coil sensitivity and then stopped by using a low-pass filter at a conveniently large frequency.

The parallel circuit between the 220 pF capacitor and 3.3 MΩ resistor captures the coil-self resonance at about 200 Hz, reducing the gain of the stage for increasing frequency above it.

The output stage provides a variable gain selectable between 1 and 100 in 20 dB steps.

**Figure 4:**
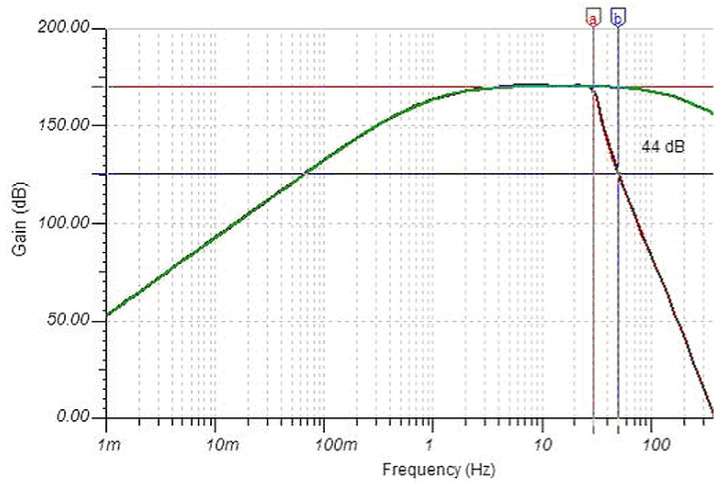
Frequency response of the induction coil sensor: without (green curve) and with (dark red curve) the 36 Hz LPF: letters “a” and “b” indicate the corner frequency and the 50 Hz point of the curve.

As shown in Figure 4, the performance is comparable to the best commercial products with an important option:

- a Chebychev 30 Hz 6-pole low-pass filter (LPF) (visible as three cascaded cells between “Filter in” and “Filter out” in Figure 3) implemented in a Sallen–Key architecture; it ensures 44 dB of attenuation at the 50 Hz disturbing frequency with a well-usable bandwidth around and above the Schumann resonance.

**Figure 5:**
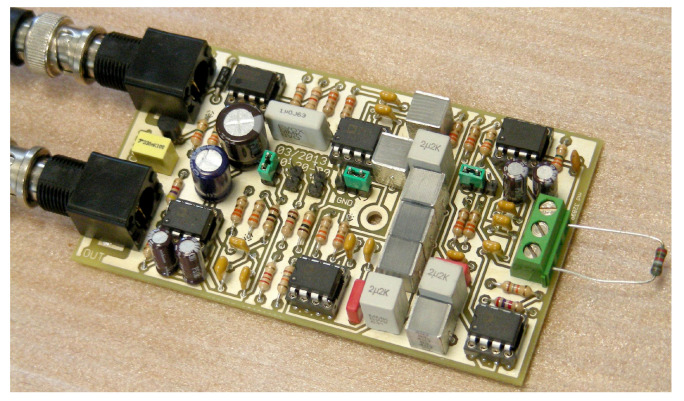
Picture of the ICS101 induction coil amplifier.

The filter uses feedback capacitors of quite a large value ( 6.8 μF and 22 μF) due to the low cut-off frequency achieved, which cannot be of the electrolytic type and are to be built with parallel connection of the largest available samples of the non-polarized type, e.g., polyester capacitors. Polyester capacitors have very linear behavior and are quite stable with respect to temperature changes. They are visible as gray blocks on the printed circuit board of Figure 5.

The characteristics of the induction coil system used as an H-field sensor are summarized in Table 3.

The reported equivalent input noise does not depend on the gain since the noise sources are all located at the input stage:

- the input voltage noise $e_{n}$ of the input Operational Amplifier (OA) itself; the selected OA was a TL071 [26] or TL081 [27] due to a matter of availability, so $e_{n}=18\;nV/\sqrt{\mathrm{Hz}}$ at 1 kHz and $e_{n}=0.4\;\mu V$ overall over the 10 Hz to 10 kHz frequency interval;
- the input current noise $i_{n}$ is quite low, being a CMOS architecture (the reason for the selection), which well matches the moderately large source impedance represented by the coil reactance and resistance (about 100 kΩ for the former, given by the 1650 H inductance at 10 Hz, and 11,220 Ω for the latter); this amounts to only 1 nV/$\sqrt{\mathrm{Hz}}$, which is negligible;
- the thermal noise of the winding resistance, $e_{R,n}$, for the said 11,220 Ω corresponds to 13.8 nV/$\sqrt{\mathrm{Hz}}$.

The overall noise is the rms composition of the two noise voltage sources $e_{n}$ and $e_{R,n}$:

(1) $$e_{tot,n}=\sqrt{e_{n}^{2}+e_{R,n}^{2}}=22.7nV/\sqrt{\mathrm{Hz}}$$

It is observed that the equivalent input magnetic noise (calculable from the equivalent noise voltage $e_{n}$ and the coil antenna factor AF as 0.1 pT/$\sqrt{\mathrm{Hz}}$ in the range 0.1–10 Hz) is better than high-performance SQUID sensors [28]. Its performance in terms of antenna factor (also called simply “gain” or “sensitivity”) are almost identical to the THEMIS search coil magnetometer [29]. The LEMI 120 coil [18] (recently used for investigations of EQs in India [30]) exhibits a similar performance down to about 0.1 Hz and can thus be considered equivalent. On the contrary, the reported performance is much better than all the compared compact high-resolution magnetometers in [31], except for the atomic magnetometer.

The expression “ferrite core” followed by “voltage gain” and “impedance increase” [32] indicates what the effect of inserting the ferrite core into the coil is: the output voltage increases (higher sensitivity), but at the same time, the coil inductance (and thus the internal impedance) increases. The latter must be duly considered when designing the input stage impedance not to deteriorate the improved sensitivity with the coil internal voltage drop.

### 2.2. Electric Field Sensor

**Figure 6:**
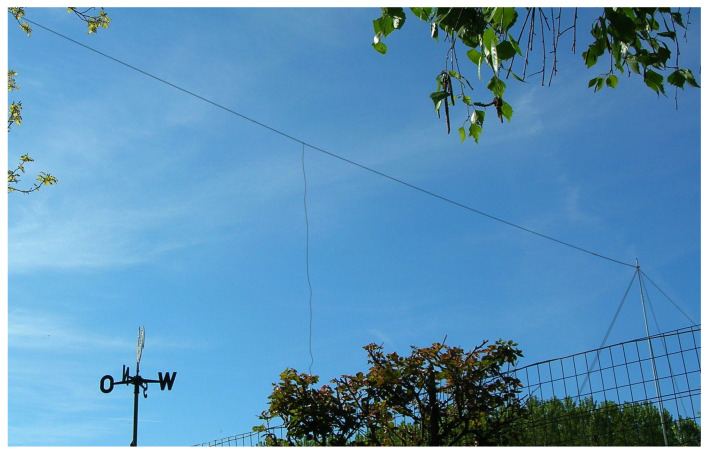
Picture of the Marconi wire antenna for E-field measurements (dimensions and construction details provided in the text).

The electric field (E-field) sensor is built around a large Marconi wire antenna using a 10-m-high T-shaped aerial with a 15 m capacitive cap (increasing the total antenna capacitance and thus reducing its input impedance), as shown in Figure 6. A spherical electrode (as commonly used for high-voltage testing) has been proposed sometimes, but it is more affected by local effects (such as wind and animals moving nearby), and the weight and lift are more critical (especially in the case of strong wind) and the overall gain is lower. The received Schumann resonance signal of the Marconi antenna is strong and stable, and the bandwidth extends down to a few tenths of Hz.

**Figure 7:**
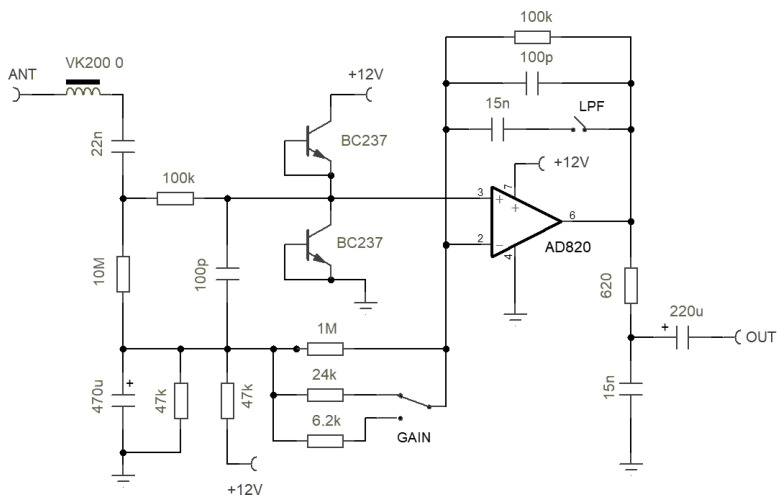
Schematic of the high-input impedance amplifier of the Marconi wire antenna.

**Figure 8:**
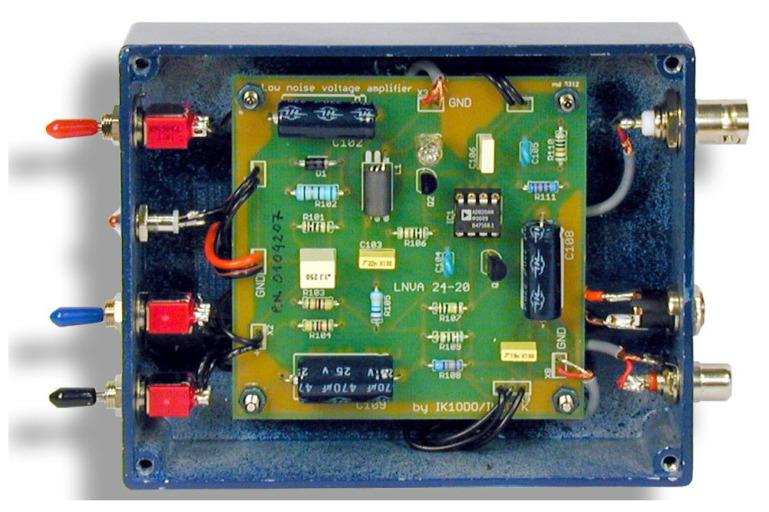
Picture of the LNVA 20–24 amplifier for the Marconi wire antenna.

A LNVA-20-24 voltage pre-amplifier, built around an Analog Devices AD 820 [33] OA and featuring a large 10 MΩ input impedance, is directly connected at the base of the antenna. The schematic is shown in Figure 7, and its realization, including the watertight metallic housing, is shown in Figure 8.

As the input signal is exposed to outdoor e.m. threats (in particular high-frequency radio pollution and overvoltages), the input line is protected against the former by a VK200 RF ferrite and by two diodes (obtained using two short-circuited BC237 transistors), clamping the potential between the two power rails of 0 V and 12 V through the 100 kΩ resistor.

Its output impedance was optimized for connection to a sound card by inserting a 620 Ω resistor and low-pass filtering the output while removing, at the same time, any DC offset by a series-connected 220 μF capacitor.

The bandwidth was defined by the combination of the 10 MΩ and 22 nF input resistor and capacitor (giving about 0.7 Hz of high-pass corner frequency) and the parallel of 100 kΩ and 15 nF in the feedback network above the OA (giving about 100 Hz of low-pass corner frequency). The latter is an option without which the bandwidth extends up to about 15 kHz.

The main characteristics of the LNVA-20-24 amplifier are summarized in Table 4.

It is observed that the selected AD820 [33] has such a low input noise current, $i_{n}$ ( 18 fApp between 0.1 Hz and 10 Hz, and 0.8 fA/$\sqrt{\mathrm{Hz}}$ at 1 kHz), it makes the voltage drop noise on the input resistor lower than the OA input noise voltage $e_{n}$, as shown below. The only relevant contributing term is then the thermal noise of the input terminating 10 MΩ resistor.

The reported equivalent input noise does not depend on the gain since the noise sources are all located at the input stage:

- the OA input voltage noise $e_{n}=25\;nV/\sqrt{\mathrm{Hz}}$ at 10 Hz, $e_{n}=100\;nV/\sqrt{\mathrm{Hz}}$ at 1 Hz, and $e_{n}=2\;\mu Vpp$ overall over the 0.1 to 10 Hz frequency interval;
- the input current noise, $i_{n}$, is quite low for a CMOS architecture (the reason for the selection): $i_{n}=18\;fApp$ overall over the 0.1 Hz to 10 Hz; this well withstands the large input and source impedance represented by the antenna reactance and the 10 MΩ resistor, resulting in about 0.18 μVpp/$\sqrt{\mathrm{Hz}}$, remaining negligible with respect to the noise voltage $e_{n}$, even including the increasing antenna reactance at lower frequencies;
- the thermal noise of the 10 MΩ input resistor corresponding to 0.41 μV/$\sqrt{\mathrm{Hz}}$ representing the largest noise contribution.

The overall noise is thus contributed almost entirely by the input resistor, except possibly at the lowest frequencies, around 0.1 Hz or so, where a slight increase could be expected.

### 2.3. Geophone

Many monitoring stations of the Opera 2015 project were also equipped with a vertical geophone, connected to a special low-noise pre-amplifier, also suitable to drive a sound card. The device is not a substitute for the seismograph but allows discriminating when ground vibrations can induce microphonic effects on the induction coil. False positives, in fact, can originate from vibrations next to the monitoring station (e.g., traffic on a road nearby, rolling noise from transportation systems or heavy construction activity), causing a microphonic effect on coils moving in the otherwise static Earth’s magnetic field.

In addition, the available geophone signal allows the identification of the arrival of seismic vibrations at the station, aligning with any microphonic effect in the H-field and possibly E-field sensors. Signals occurring between the occurrence of the EQ and its arrival at the station are caused by the piezoelectric effect and, in general, electro-seismic effects, at the originating seismic fracture or along the path (traveling faster than the acoustic waves).

**Figure 9:**
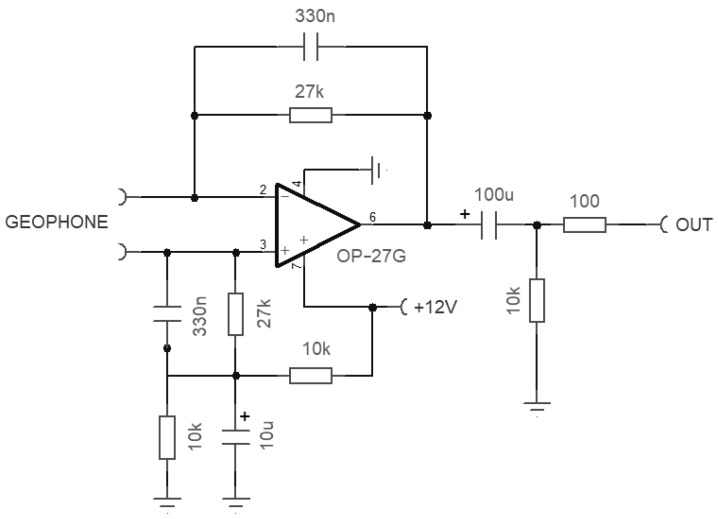
Schematic of the geophone amplifier.

**Figure 10:**
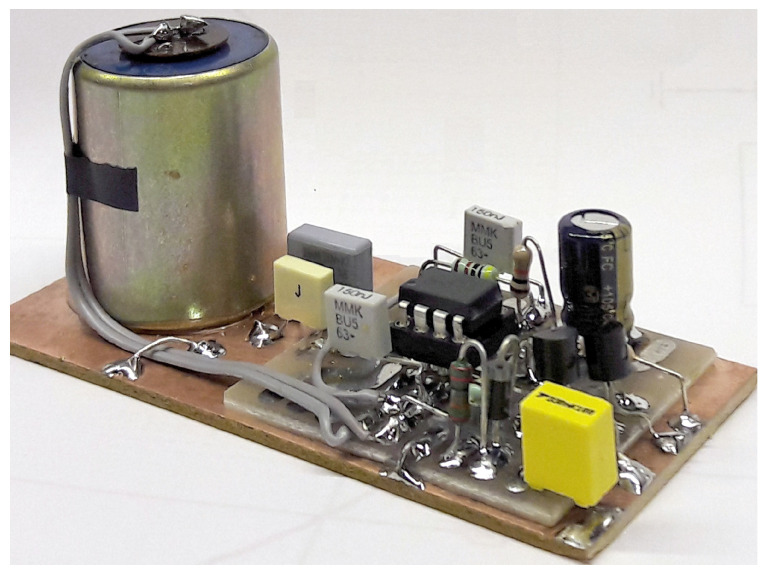
Picture of the geophone (left side) with an amplifier. Shielding and ground plane distribution were achieved using a copper board on which a second printed circuit board was located and electrically bonded where needed.

The geophone coil resistance is 375 Ω, and the circuit is electrically floating. The signal is measured with a floating OA circuit built around an OP 27 [34], which is shown in Figure 9. The combined frequency response of the geophone (high-pass behavior) and pre-amplifier (band-pass between 0.15 and 12 Hz is also shown in Figure 9). The geophone vertical element (shown mounted on a PCB in Figure 10) is a SM-4/UB8 manufactured by I/O Sensor [35], with a sensitivity of 28.8 V/(m/s). The damping factor of each unit is calibrated and brought with a tuning shunt resistor to a value of 0.6 to 0.7 (slightly resonating below the critically damped threshold value of 0.707).

The amplifier (whose schematic is shown in Figure 9) implements a transconductance architecture through the two 27 kΩ resistors. The added 330 nF capacitors give a cut-off frequency of 17.9 Hz, providing a pole in addition to that of the natural frequency response of the geophone and drastically reducing the total noise and interference from the usual 50 Hz disturbance.

### 2.4. Data Acquisition System

Audio cards were used for data acquisition and computer interface in order to keep down costs while keeping high quality, not only in terms of vertical resolution but of noise rejection. Almost all sound cards indicate a working frequency interval of 20 Hz to 20 kHz, but, in reality, many of these products have a workable interval extended down to a few Hz with tolerable gain reduction. Products such as the Creative SB0270 and the Creative SB1095 were used successfully for the recording of geomagnetic pulsations down to about 0.1 Hz. Attenuation could be simply caused by DC-blocking input capacitors, which can be easily replaced with larger ones, providing an immediate frequency extension. These cards provide high-quality recording with 24-bit resolution and 96 kHz sampling.

Presently there are portable oscilloscopes and data acquisition systems available at almost the same price as a sound card, and the previously discussed measuring systems with their amplifiers can all fit well, with some care for the vertical range (that is, not all such acquisition systems have the sensitivity of former sound cards and their input ranges tend to be located on the high side). The provided selectable gain can still well accommodate dynamic ranges in the hundreds of mV and Volts.

### 2.5. Software

The software used for analysis and display is based on a public domain program called SpectrumLab [36]. Many features of this program are well suited for the processing of the acquired signals: calculation and display of spectrograms (by Short Time Fourier Transform, STFT) in various forms, the direction of arrival (applied to the signals from the orthogonal induction coils), visual comparison of different signals with informative graphical presentation and controlling the recording through the audio cards. SpectrumLab, besides analyzing the data in real time providing the spectra for the website, allows a time-stamped archive of spectrograms and original audio files. The program is particularly useful for processing real-time data without losing displayed information, even when changing parameters on the fly.

Of course, data can be imported into any mathematical or signal processing environment, such as Matlab, where routines can be implemented. The selection of smart classification criteria and metrics (that support the rejection of extraneous signals enhancing the components supposedly originating at the EQ) will be the next step of the research, exploiting the large Opera2015 dataset.

SpectrumLab was extensively used during 2015 and in the subsequent years to process and inspect acquired data, looking for the repetition of patterns, preferring a visual critical comparison rather than applying blind, although mathematically more accurate, similarity criteria. One of the reasons was the possibility of increasing our knowledge of the external sources of, e.g., human-made noise, improving the evaluation of the false precursors. Collected data, as well as the output of our spectrum analysis, have been recently used for comparison in relation to the Tonga event that occurred on 15 January 2022 [16].

## 3. Dataset of Recorded Events

A significant set of data was recorded during 2015 and is still available at www.vlf.it [15]. EQ events of relevant intensity all occurred in different parts of the world with a significant distance from the monitoring stations, but some events of moderate magnitude between 3 and 4 occurred at various locations in Italy, which happened to be close to at least one of the monitoring stations, as is the case for Sicily. Out of the anticipated 15,532 confirmed EQ events monitored during 2015, a selection of the most relevant ones in terms of detectability is provided in Table 5. The following criteria are those of a sufficiently large intensity paired with a reduced distance (most of the EQs are from Italy and the Mediterranean sea) and the absence of confounding co-signals that could impair the identification of precursors. In addition, the listed events are available from multiple stations so that they can be selected based on different criteria, such as always the highest signal from the nearest station or selecting the station with the lowest natural and human-made noise (e.g., based on statistics of previous months).

To the aim of verifying detectability and testing the radio seismic indicator of Section 5, positive findings of EQ precursors were taken from the literature for some significant events of the last 25 years, as shown in Table 6.

The events listed in the two tables span between 3.2 and 9.0 in magnitude and between a few km up to about 14,000 km between the epicenter and station. The tables report the location and the date with UTC time together with the bibliographic reference for the events taken from the literature.

## 4. Signals and Post-Processing

**Figure 11:**
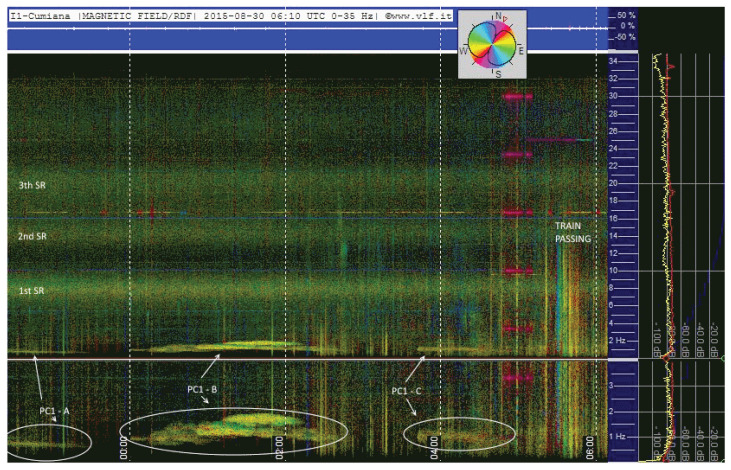
Spectrograms from two orthogonal induction coils; signals processed with the direction-finding technique (spectrum color indicating the direction of arrival).

In this section, examples of STFT-based data processing and visual interpretation are provided for the signals captured from the discussed electric and magnetic sensors and geophone. Figure 11 shows an example of the direction-finding techniques exploiting the two orthogonal coils and giving a color-coded direction of arrival.

**Figure 12:**
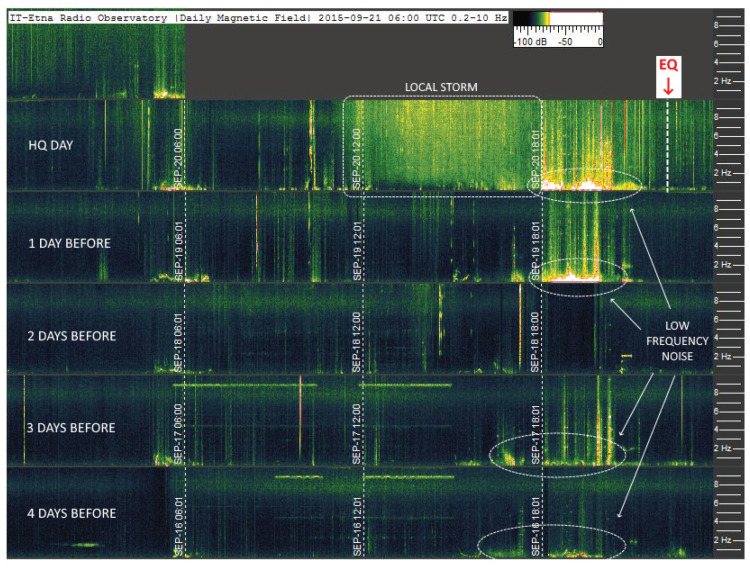
Rolling spectrograms of the four days preceding event no. 034 (Table 5).

In Figure 12, there is a clear example of an EQ event (event 034 in Table 5) preceded by a widespread increase in background noise in the previous 10 hours and an even stronger increase in the last 3 hours, returning to normal levels after the EQ. A cross-comparison with the data provided by Blitzortung [49], however, indicates a concomitant lightning activity, confirmed by comparison with observations of the previous days: this casts some doubts on the presence of detectable precursors in such and similar cases.

In a year of monitoring we could capture two Etna eruptions with recording devices located just a few km from the volcano’s vents; see events 004 and 044 in Table 5. The eruption was observed from its inception, with the geophone informing us constantly of the lava flow rising to the surface, recording vibrations as a weak continuous EQ lasting tens of hours. However, even in these two cases, the absence of very low-frequency signals before and during the eruption was clear. No abnormal signals nor variation in Schumann resonances were observed. The trace of the eruption vibration is very clear on the geophone spectrogram, while in the induction coil spectrogram, nothing appears in addition to the Schumann Resonances and easily recognizable anthropogenic noise.

In general, a deep and extensive analysis was carried out daily with cross comparisons between stations, looking for the periodic recurrence of suspicious patterns and the repetition of these patterns in other EQs. A lot of false local signals have been identified and excluded, including the effects of wind, storms, etc., besides human-made noise. In other words, although at first glance there are many acquisitions showing the presence of possible EQ precursors, after verification, these signals are found to be non-repetitive and not systematically matched with seismic events.

The verification methods were kept simple, using mainly basic Fourier spectrum analysis of the recorded electromagnetic waves and comparing them to external sources of information, such as Blitzortung for thunderstorms and the lighting activity, and other observatories to access similar recordings at different distances and with different instrumentation (such as the Kiruna Atmospheric and Geophysical Observatory [50] or the Sodankylä Geophysical Observatory [51]). A significant deal of work was carried out analyzing the same location and the occurrence of similar spectral patterns in quite different periods of time, thus showing repetition and not uniqueness related only to the occurrence of EQ events.

**Figure 13:**
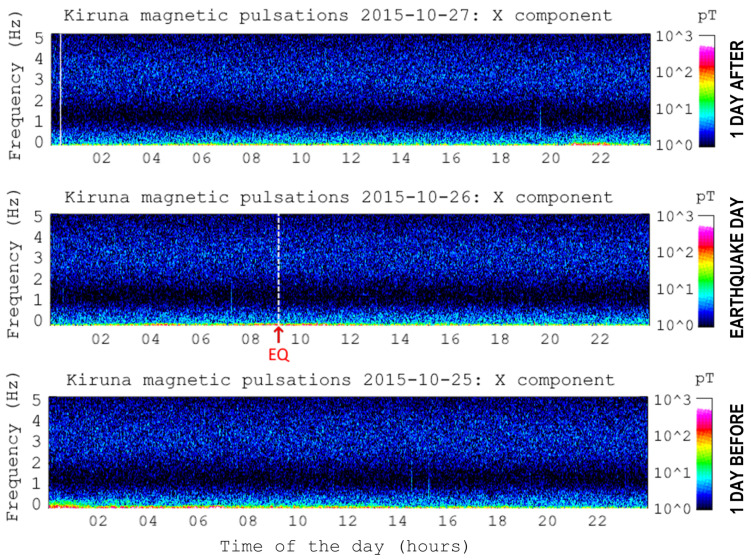
Rolling spectrograms for the interval ±1 day around event no. 036 (Courtesy of the Kiruna Atmospheric and Geophysical Observatory [50]).

A visual confirmation that global detectability has not been achieved so far is given in Figure 13, where event no. 036 from Table 5 (26 October 2015) is shown for what was recorded at the Kiruna Geophysical Institute (Sweden). The EQ event marked in red is preceded by one day of quiet spectrum, which does not show the electromagnetic activity of several nT reported in [52]. It is, nevertheless, recognized that there are periods of intense geomagnetic activity that could mask the absence of precursors on a global scale, as it is for another period of 2015 around mid-September, also impacting event no. 031 of Table 5.

## 5. Radio Seismic Indicator

The source of emissions at the EQ has often been modeled as a vertical electric dipole [53] for which equations of field propagation can be laid down. In particular, for the transverse magnetic mode, a nearly quadratic law with distance can be determined. The thorough solution of the Helmholtz potential equations with boundary conditions gives place to a set of equations for the non-null components, namely (in spherical coordinates) the electric field along coordinates *r* and θ ($E_{r}$ and $E_{\theta }$), and, as anticipated, the magnetic field along ϕ ($H_{\phi }$). Li and Pan [54] proposed accurate formulations, specifically valid for the low-frequency interval of EQ emissions in the reactive region. However, depending on frequency and distance, radiative and reactive terms at the receiver position may mix to a different extent. Such terms are characterized by a linear, and quadratic exponent with respect to distance; a third-order term is often accompanying the first magnetic field reactive region. An exact intermediate exponent value of 1.5 for *D* was selected as a compromise, providing a proportionality of 30 dB of intensity change for each 20 dB in distance.

The intensity of the source, then, is considered equal to the square root of the EQ power (the EQ energy measured by the magnitude using an empirical approximation of $10^{1.5M}$, assumed concentrated in the short time interval when the maximum EQ intensity takes place). Putting together such a measure of EQ power with the assumed propagation law for the $H_{\phi }$ component, the following RI (radio seismic indicator) expression was derived.

(2) $$RI=20\log_{10}\left(\frac{\sqrt{10^{1.5M}}}{D^{1.5}}\right)$$

where *M* is the EQ magnitude (in Richter scale units) and *D* is the distance (in km) on the Earth’s surface between the EQ event and the observer.

Considering the epicenter in the place of the hypocenter, thus neglecting the depth at which the EQ event occurred, is an acceptable approximation, considering that in most cases the depth is (much) less than the horizontal distance (for example, only EQ event no. 005 in Table 5 violates this assumption and is characterized, in any case, by a sufficiently large RI value). It may be said as well, that this approach considers only the surface distance has been commonly used to derive the other indicators available in the literature, such as Molchanov et al. [55] and Hattori et al. [39].

**Figure 14:**
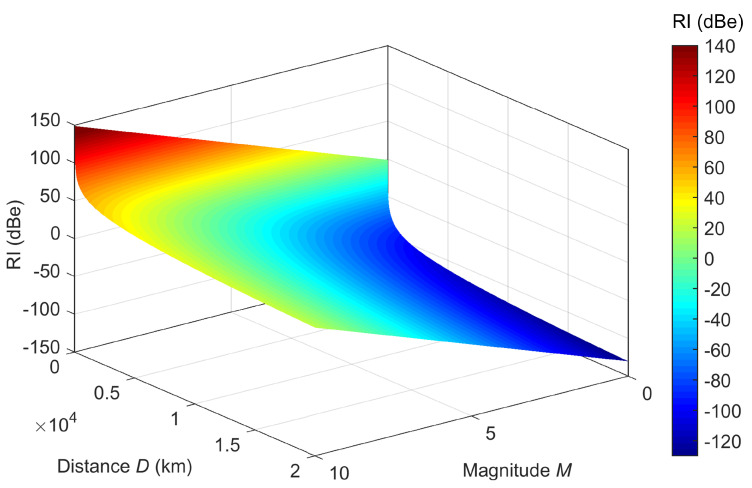
Graphical representation of RI vs. distance, *D*, and magnitude, *M*.

RI is conventionally expressed in dBe, with “e” standing for “earthquake”: we set RI=0 dBe for an EQ with magnitude M=4 and with an epicenter located D=100 km distance from the observer. RI is plotted in Figure 14.

**Figure 15:**
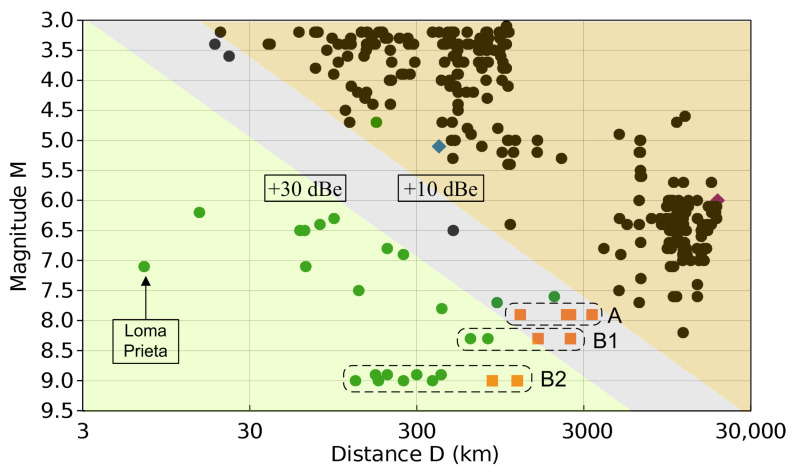
Magnitude–distance plot of collected EQ events: Opera 2015 (Table 5, black), positively identified EQs from the literature (Table 6, green), dubious positive EQ events (blue), dubious negative EQ events (orange) and [52] (purple). The light green and light brown areas correspond to RI≥30 dBe (positive precursor) and RI≤10 dBe (negative precursor) with a gray area in between.

Figure 15 reports 316 EQ events, 280 of which are from the Opera Etna Volcano station and the other 36 are from scientific papers (already listed in Table 6). It is clear that positive results are almost exclusively located in the green area for RI≥+30 dBe. The gray area, with RI between +30 and +10 dBe, contains mixed results, where the advantages of a specific post-processing method can make the difference. The light brown area with RI≤+10 dBe is characterized almost completely by negative radio seismic detection.

There is one event reported as positive, but based on the total electron count criterion and not on electromagnetic precursors [44], marked as a light blue diamond. There is also one positive event falling into the light brown area of RI≤10 dBe.

Some intense EQ events are then reported as both positive and negative (so with and without precursors) and are marked by “A”, “B1” and “B2” in Figure 15: “A” refers to dubious detections, where a near station cannot detect precursors, and is reported instead by another station farther away; “B1” and “B2” show the same EQ event for stations at different distances, with the last ones (in orange) falling in or toward the grey area, justifying the lack of an identified precursor.

One paper [52] then stands out for reporting a positive identification with one of the largest distances between the EQ and station, located deep in the negative identification area, which casts doubts on the correctness of the results (it is marked in purple, located at the right end).

Despite the fact that southern Italy is an active seismic zone, examining Figure 15, no Opera 2015 recorded event falls into the minimum distance/intensity range to be in a condition for receiving precursors, and, in fact, we could not identify any significant e.m. activity that unequivocally can be put in relation with the EQs recorded by the Istituto di Vulcanologia. Conversely, the main EQ event, to which most of the articles on radio seismic precursors refer, is the Loma Prieta EQ in 1990: the calculated RI exceeds +80 dBe, and the monitoring station was positioned 7 km away from the epicentre on an estimated 40-km long fault line, and exactly above the break line.

In conclusion, although the six Opera 2015 stations were equipped with highly sensitive devices and placed in strategic locations, we have not detected any signals corresponding to local EQs. Those minimum conditions for RI never happened, although in a few cases, we see that events are positioned at the margin of the grey zone.

## 6. Conclusions

This work has described the set of instruments designed and built for the Opera 2015 project in Italy, where six monitoring stations were deployed, interconnecting the data acquisition systems to form an integrated dataset of results [15]. Two other stations at the EGO-Virgo experiment and the Sos Enattos site were then deployed in successive years.

The acquired data were subjected to spectral analysis, including direction finding, complementing the analysis with an as comprehensive as possible evaluation of extraneous sources in order to remove false positive events. The considered methods were the verification of thunderstorm and lightning activity [49] to exclude a large portion of intense perturbations of the lower frequency interval, followed by the analysis of the repetitiveness across different days in the same or similar time of the day, accumulating visual statistics of natural and human-made noise (that need long observation times as there is no pre-determined behavior or reference statistic).

Performances of designed and built instruments are aligned with the state-of-the-art published in the scientific literature or commercially available, as discussed in Section 2. The design schematics that were discussed in Section 2 allow readers to realize similar instruments, achieving a significant level of performance in terms of sensitivity, usability and cost.

From a general viewpoint, the results of the activity on the acquired data can be summarized as follows:

- a radio-seismic indicator (such as the RI defined in this work and used in the Opera 2015 project) is useful to establish the possibility of receiving and recognizing the precursors, assigning a weight to each event, as studying all the earthquakes on a global scale is useless;
- the minimum condition necessary for the detection of a precursor, using our method, comes with an RI value of at least 30 dBe;
- for these reasons, radio seismic precursors cannot be detected on a global scale, even for medium-intensity earthquakes with a magnitude of up to 6;
- a monitoring station located in an urban area has almost no chance of detecting radio precursors due to the abundance of human-made noise sources, which are also quite variable;
- it is unlikely, in light of these considerations, that the general prediction of earthquakes could be imminent unless monitoring stations happen to be very close to the affected area.

The network of monitoring stations set up for Opera 2015 is still active with four main active stations and has provided useful recordings for other studies related to recent earthquakes (Tonga on the 15 January 2022). Forthcoming activity is the intensification of such collaborations and the automation of access to the database of raw data, which is so far manual.

## Figures and Tables

**Table 1:** Opera 2015 monitoring stations.

Station	Latitude	Longitude	Height (m)	Measurement Equipment
IK1QFK, Cumiana (TO), Italy (CMN)	44.956387	7.419848	278	Two orthogonal induction coils, Marconi antenna, geophone, GPS
CSP VLF, Rifugio Pontese (TO), Italy (PNT)	45.495899	7.368851	2182	Multi-turn aerial loop, diff. E-field receiver
Etna Radio Obs. Nicolosi (CT), Etna Park, Sicily, Italy (ERO)	37.631177	15.022294	835	Induction Coil, Geophone
Romagna Obs., Fiumicino (FC) Sogliano al Rubicone (FC), Italy (SGL)	43.938898	12.226351	543	Induction Coil, Geophone
Northern Cross Radiotelescope, Medicina (BO), Italy (IRA)	44.524359	11.644978	7	Induction coil, static electric field receiver
TORINO VLF Monitoring Station, Turin, Italy (TUR)	45.066278	7.723683	246	Marconi antenna, diff. E-field receiver, flux-gate sensor

**Table 2:** Earth’s magnetic field parameters of monitoring stations: latitude and longitude of the dipole and quasi-dipole models; declination, D, inclination, I, North oriented intensity, X, Eastern oriented intensity, Y, horizontal intensity, H, vertical intensity, Z, and total intensity, F, as main field components and secular variation (expressed per year) reported in parentheses.

Station	Dip. Lat.	Long. Dip.	Quasi-Dip. Lat.	Quasi-Dip. Long.	D °East	I °Down	X (nT)	Y (nT)	H (nT)	Z (nT Down)	F (nT)
CMN	45.82	89.76	39.53	83.09	1.787 (0.148)	60.923 (0.01)	22,856 (12.1)	713 (59.5)	22,867 (14.0)	41,124 (41.3)	47,054 (42.9)
PNT	46.36	89.90	40.17	83.12	1.768 (0.15)	61.451 (0.01)	22,538 (11.6)	696 (59.4)	22,549 (13.4)	41,446 (41.5)	47,183 (42.8)
ERO	37.41	95.03	31.03	89.13	2.995 (0.118)	53.267 (0.017)	26,891 (14.1)	1407 (56.2)	26,928 (17.0)	36,083 (45.5)	45,023 (46.7)
SGL	44.03	94.14	38.36	87.15	2.829 (0.138)	60.243 (0.015)	23,317 (9.4)	1152 (56.7)	23,345 (12.2)	40,834 (46.9)	47,037 (46.8)
IRA	44.69	93.76	39.04	86.71	2.731 (0.14)	60.793 (0.015)	23,005 (9.2)	1097 (57.0)	23,031 (11.9)	41,197 (46.5)	47,197 (46.4)
TUR	45.88	90.10	39.66	83.37	1.859 (0.148)	61.054 (0.01)	22,788 (11.8)	740 (59.3)	22,800 (13.7)	41,224 (41.8)	47,109 (43.2)
SOS	41.09	90.35	34.16	84.35	2.170 (0.132)	56.194 (0.013)	25,373 (15.3)	961 (58.7)	25,391 (17.5)	37,920 (40.8)	45,635 (43.7)
VRG	44.02	92.36	37.97	85.60	2.466 (0.14)	59.798 (0.013)	23,535 (11.1)	1013 (57.8)	23,557 (13.5)	40,471 (44.6)	46,828 (45.4)

**Table 3:** Characteristics of the induction coil system using the ICS101 amplifier.

Parameter/Characteristic	Value
Inductance (without and with ferrite core)	23 H, 1650 H
Resistance	11,220 Ω
Lower cut-off frequency	1.3 Hz
Self resonating frequency	200 Hz
Parasitic capacitance (calculated from resonance)	385 pF
Input equivalent noise	22.7 nV/$\sqrt{\mathrm{Hz}}$
Antenna factor AF at center band ( 10 Hz)	0.22 mV/nT at gain = 0 dB
Ferrite core voltage gain	144
Ferrite core impedance increase	71

**Table 4:** Characteristics of the LNVA 20–24 amplifier.

Parameter/Characteristic	Value
Frequency range	0.7 Hz–15 kHz
Gain (selectable)	0 dB, 12 dB, 24 dB
Low-pass filter (selectable)	100 Hz, 15 kHz
Input impedance	10 MΩ
Output impedance	620 Ω
Equivalent input noise	0.4 μV/$\sqrt{Hz}$

**Table 5:** Selected EQ events of Opera 2015.

ID	Date	UTC	Location	*M*	Dist. (km)	Depth (km)	RI (dBe)
001	10 Jan. 2015	23:50:02	Sicilia sea, Italy	3.9	243	20	−13.1
002	23 Jan. 2015	06:51:20	Pistoia, Italy	4.3	793	10	−22.5
003	28 Jan. 2015	15:54:37	Creete, Greece	5.2	974	20	−11.7
004	31 Jan. 2015	06:30:00	Etna eruption, Italy	—	—	—	—
005	6 Feb. 2015	08:52:25	Lipari island, Italy	4.7	119	256	8.2
006	13 Feb. 2015	18:59:16	Northern Mid-Atlantic	6.8	3962	17	-5.9
007	17 Feb. 2015	19:42:53	Firenze, Italy	3.9	789	8	−34.4
008	4 Mar. 2015	00:00:04	Florence, Italy	3.7	739	9	−30.6
009	27 Mar. 2015	23:34:54	Creete, Greece	5.4	1056	74	−9.7
010	29 Mar. 2015	10:48:46	Calabria, Italy	3.6	116	10.7	−7.9
011	29 Mar. 2015	23:48:30	Papua New Guinea	7.6	14,372	20	10.7
012	1 Apr. 2015	04:58:11	Forlì, Italy	3.2	780	21	−38.8
013	11 Apr. 2015	05:33:13	Alpi Cozie, Italy	3.2	1035	10	−42.4
014	16 Apr. 2015	18:07:43	Creete, Greece	6.4	1090	19	4.9
015	20 Apr. 2015	01:07:42	Etna, Italy	3.6	23	2.7	13.4
016	24 Apr. 2015	15:02:54	Forlì, Italy	4.0	781	22	−26.8
017	25 Apr. 2015	06:11:26	Nepal	7.7	6446	10	1.22
018	12 May 2015	07:05:20	Nepal	7.3	6592	10.2	−5.1
019	24 May 2015	06:00:33	Aspromonte, Italy	3.9	96	62	−0.9
020	29 May 2015	13:07:57	Adriatic sea, Italy	4.2	596	15	−20.3
021	30 May 2015	11:23:02	Bonin Islands	7.6	10,795	675.4	−7.0
022	9 Jun. 2015	01:09:03	Greece	5.1	736	10	−9.5
023	9 Jun. 2015	21:49:49	Creete, Greece	5.4	1087	34.2	−10.1
024	22 Jul. 2015	12:57:43	Bologna, Italy	3.7	804	22	−31.7
025	2 Aug. 2015	06:58:06	Cosenza, Italy	4.0	206	240	−9.4
026	3 Aug. 2015	07:27:48	Cosenza, Italy	4.0	212	26	−9.8
027	24 Aug. 2015	03:43:54	Forlì-Cesena, Italy	3.5	781	9	−34.3
028	29 Aug. 2015	18:47:03	Slovenia-Italy	4.0	973	7	−29.6
029	10 Sep. 2015	07:32:08	Turin, Italy	3.1	1034	11	−43.9
030	13 Sep. 2015	01:04:34	Florence, Italy	3.8	737	9	−29.0
031	16 Sep. 2015	22:54:33	Near coast of Chile	8.2	11,808	9	0.8
032	18 Sep. 2015	19:24:52	Pesaro-Urbino, Italy	3.5	705	10	−33.0
033	19 Sep. 2015	07:12:47	Pesaro-Urbino, Italy	3.7	704	7	−29.9
034	20 Sep. 2015	22:27:58	Siracusa, Italy	3.8	74	23	0.9
035	20 Oct. 2015	10:35:50	Modena, Italy	3.5	872	7	−35.7
036	26 Oct. 2015	09:09:32	Hindu Kush, Afghanistan	7.5	4878	193	1.9
037	1 Nov. 2015	07:52:03	Slovenia-Croatia	4.8	918	10	−16.9
038	6 Nov. 2015	04:03:04	France-Italy	3.8	1031	11	−33.4
039	17 Nov. 2015	07:10:08	Greece	6.5	496	10	16.6
040	17 Nov. 2015	08:33:46	Greece	5.3	494	34	−1.3
041	18 Nov. 2015	12:15:39	Greece	5.0	509	10	−6.2
042	20 Nov. 2015	05:12:24	Greece	5.0	485	10	−5.6
043	24 Nov. 2015	22:50:54	Peru-Brazil	7.6	10,334	624	−6.4
044	8 Dec. 2015	09:28:31	Catania, Italy ${}^{\left(1\right)}$	3.4	19	2	12.9
045	20 Dec. 2015	09:46:03	Sicilian Coast, Italy	4.2	150	5	−2.3

^(1)^ Etna eruption occurred at the same time.

**Table 6:** Overview of EQ events collected from the literature.

ID	Date	UTC	Location	*M*	Dist. (km)	RI (dBe)	Ref.
E01	7 Dec. 1988	11:41	Spitak, Armenia	6.9	140	39.1	[37]
E02	17 Oct. 1989	4:15	Mt. Loma Prieta, California	7.1	52	55.0	[6]
E03	17 Oct. 1989	4:15	Mt. Loma Prieta, California	7.1	7	81.1	[6]
E04	8 Aug. 1993	8:34	Guam, Japan	8.0	65	52.1	[37]
E05	17 Feb. 1996	5:59	Biak, Indonesia	8.2	80	65.9	[3]
E06	26 Mar. 1997	8:31	Kyushu, Kagoshima region, Japan	6.5	64	43.3	[38]
E07	13 May 1997	5:38	Kyushu, Kagoshima region, Japan	6.3	64	40.3	[38]
E08	3 Sep. 1998	7:58	Iwate-ken Nairiku-Hokubu, Japan	6.1	15	56.2	[39]
E09	21 Sep. 1999	17:47	Chi-chi, Taiwan	7.6	2000	15.0	[40]
E10	23 Oct. 2004	8:56	Chuetsu, Niigata, Japan	6.8	250	30.1	[40]
E11	8 Oct. 2005	nd	Muzaffarabad, Kashmir, Pakistan	7.7	908	26.8	[37]
E12	15 Nov. 2006	nd	Kuril Island, Japan	8.3	2520	11.2	[41]
E13	15 Nov. 2006	nd	Kuril Island, Japan	8.3	750	19.1	[41]
E14	15 Nov. 2006	nd	Kuril Island, Japan	8.3	1540	14.4	[41]
E15	13 Jan. 2007	nd	Kuril Island, Japan	8.1	850	33.6	[41]
E16	13 Jan. 2007	nd	Kuril Island, Japan	8.1	1630	25.1	[41]
E17	13 Jan. 2007	nd	Kuril Island, Japan	8.1	2609	19.0	[41]
E18	6 Mar. 2007	nd	Singkarak, Sumatra	6.4	79	39.1	[42]
E19	25 Mar. 2007	0:41	Noto-Hantou peninsula, Japan	6.9	200	34.5	[43]
E20	4 Oct. 2007	nd	India/Pakistan/Nepal area	4.6	628	−14.9	[44]
E21	25 Nov. 2007	nd	India/Pakistan/Nepal area	4.7	172	3.4	[44]
E22	12 May 2008	6:28	Wenchuan, China	7.9	1251	25.6	[45]
E23	12 May 2008	6:28	Wenchuan, China	7.9	2376	17.2	[45]
E24	12 May 2008	6:28	Wenchuan, China	7.9	2496	16.6	[45]
E25	12 May 2008	6:28	Wenchuan, China	7.9	3368	12.7	[45]
E26	6 Apr. 2009	3:32	L’Aquila, Italy	6.3	630	10.5	[3]
E27	16 Aug. 2009	nd	Mentawai, Sumatra	6.7	208	31.0	[42]
E28	9 Sep. 2009	nd	Tasikmalaya, Indonesia	7.5	135	48.6	[46]
E29	30 Sep. 2009	nd	Padang, Sumatra	7.6	114	38.8	[42]
E30	25 Oct. 2010	nd	Mentawai, Sumatra	7.8	424	38.2	[42]
E31	11 Mar. 2011	5:46	Tohoku, Japan	8.9	170	66.6	[47]
E32	11 Mar. 2011	5:46	Tohoku, Japan	8.9	200	64.5	[47]
E33	11 Mar. 2011	5:46	Tohoku, Japan	8.9	300	59.2	[47]
E34	11 Mar. 2011	5:46	Tohoku, Japan	8.9	420	54.8	[47]
E35	11 Mar. 2011	5:46	Tohoku, Japan	9.0	301	60.6	[48]
E36	11 Mar. 2011	5:46	Tohoku, Japan	9.0	642	50.8	[48]
E37	11 Mar. 2011	5:46	Tohoku, Japan	9.0	1295	41.6	[48]

## Data Availability

Not applicable.
